# PSEUDOEPITHELIOMATOUS, KERATOTIC, AND MICACEOUS BALANITIS

**DOI:** 10.4103/0019-5154.62753

**Published:** 2010

**Authors:** P S Murthy, Kusumika Kanak, Leena Raveendra, Pallavi Reddy

**Affiliations:** *From the Department of Dermatology and STD, Vydehi Institute of Medical Sciences and Research Center, Whitefield, Bangalore-560 066, India.*

**Keywords:** *Buschke-Lowenstein tumors*, *gaint condyloma*, *keratotic and micaceous balanitis*, *penile horn*, *penile malignancy*, *pseudoepitheliomatous*

## Abstract

A 51-year-old circumcised male presented with hard, thick, keratotic, nail-like covering of the skin of his glans penis of 2 year duration. Histology showed acanthosis, papillomatosis, and elongated rete ridges into the dermis suggestive of pseudoepitheliomatous, keratotic, and micaceous balanitis with features of cellular atypia. Partial penile amputation was done. There was no recurrence after 6 months of follow up.

## Introduction

Pseudoepitheliomatous, keratotic, and micaceous balanitis (PKMB) is an extremely rare penile disorder involving the skin of the glans that occurs in older men who undergo circumcision late in life. PKMB was first described by Lortat-Jacob and Civatte in 1966 in the French literature.[1] This condition is considered pseudomalignant, premalignant, or as a low grade squamous malignancy.[2] PKMB along with penile horn and giant condyloma (Buschke-Lowenstein tumors) is a group of tumors which initially show benign histology or later show either a low grade or delayed malignant growth potential.[3]

## Case Report

**Figure 1:**
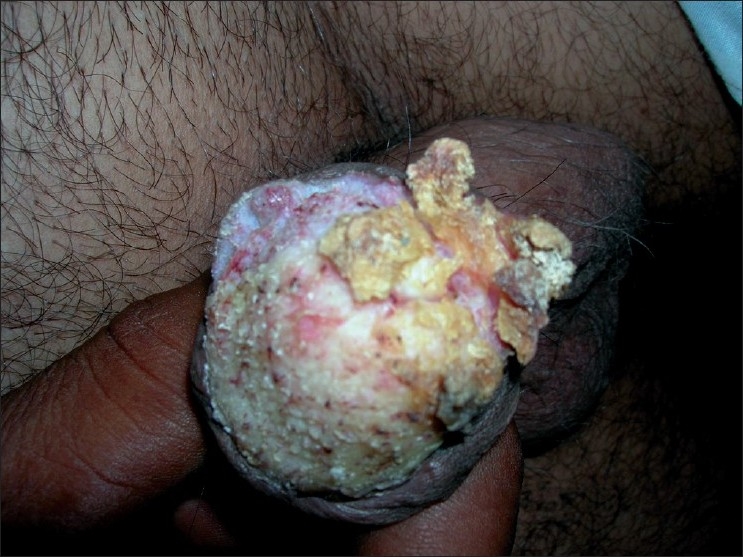
Pseudoepitheliomatous, micaceous, and keratotic scales on a circumcised penis

**Figure 2:**
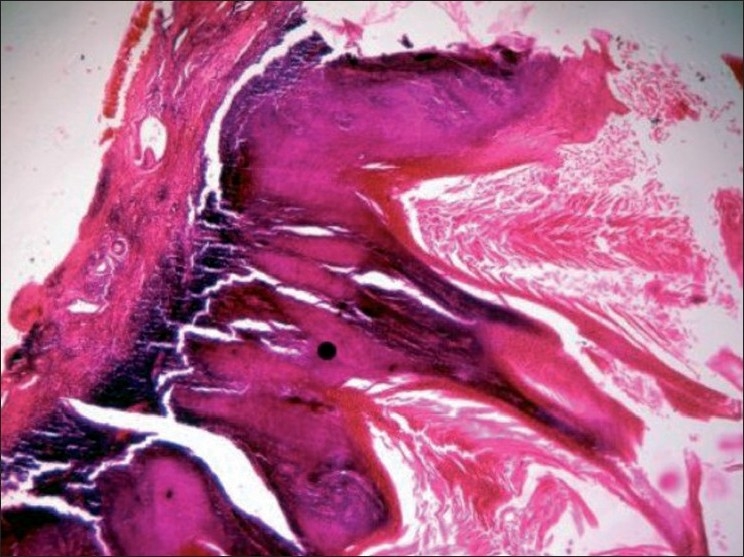
HPE showing hyperkeratosis, papillomatosis, and invasiveness of the lesion (40×)

A 51-year-old man presented with progressively increasing thick, hard, warty lesions over the penis of 2 year duration [Figure 1]. One year back, he underwent circumcision for phimosis, and a biopsy from the lesion then showed hyperkeratosis and papillomatosis without any invasive features. At the time of presentation now, clinical examination revealed a thick, hard, warty, nail-like, pellicle covering the whole of the glans penis. In some areas mica-like scales were visible with free edges. The surface was rough with warty projection. Penile skin was dry and inelastic. Some of the keratotic tissue could be peeled off with difficulty without bleeding. Cracking, fissuring, and ulceration were seen at places on the surface of the lesion. The penile shaft, scrotum, and inguinal regions were normal. There was no regional lymphadenopathy. Histopathological examination revealed acanthosis, prominent papillomatosis, elongated rete ridges forming “pushing margins,” and features of cellular atypia [Figure 2]. Dermis showed a dense band of lymphocytic infiltrate, focally obscuring the dermo-epidermal junction.

In view of the early evidence of the invasive nature histologically, therapeutic partial amputation was done. The patient was followed up for 6 months without recurrence or lymphadenopathy.

## Discussion

PKMB was first named and described by Lortat-Jacob and Civatte in 1966 in the French literature and by Bart and Kopf in 1977 in the English Literature.[4] Only a few cases have been reported since then in India. This rare condition is mostly reported in elderly males with phimosis and is considered as a form of pyodermatitis or pseudoepitheliomatous response to infection. The keratotic scales is usually micaceous and resembles psoriasis.[1] Most patients are over the age of 50 and are frequently circumcised later in life, but has also been reported in an 18 year old boy.[5] In one of the cases, crust was kept in 10% potassium hydroxide solution in the test tube overnight and was found to have dissolved totally showing that it was nothing but keratin.[6] Though originally considered to be a benign entity, it has been shown to be capable of invasive growth by Bart and Kopf who considered the lesion to be in intermediate stage between benign hyperplasia and squamous cell carcinoma.[4] However, the histological spectrum can range from hypertrophic-hyperpalastic penile dystropy to verrucous carcinoma, so much so that a new name, micaceous and verrucous malignant balanitis has been suggested for this disorder.[7] Despite diligent search for a viral etiology, human papilloma virus (HPV) has not been demonstrated and its role in pathogenesis or its transformation to verrucous carcinoma has been confirmed.[8]

The treatment of PKMB should be conservative when there is no histological evidence of malignancy.[3] All such patients should be followed up. Some lesions have been treated with topical 5-flurouracil cream,[1,2] with cryotherapy, and shaving biopsy plus electrocoagulation only to relapse.[9] In one of the studies, triamcinolone acetonide cream (0.1%), 5-fluorouracil (1%), and podophylline application (20%) were tried one after other without benefit.[5] Whenever there is cellular atypia, surgical excision produced excellent cosmetic and functional results.[7] When frank malignancy is seen, excision with wide margin is the rule.[3]

In our case, when first seen, initial histology was benign, but subsequently after follow-up, some element of invasiveness along with cellular atypia were seen. Hence, partial amputation of penis was done. The aim of reporting this case is to emphasize the importance of long-term follow-up in cases of PKMB.

## References

[1] James WD, Berger TG, Eliston DM (2006). Andrew's Disease of the skin. Name of the book missing.

[2] Krunic AL, Djerdj K, Starcevic-Bozovic A, Kozomara MM, Martinovic NM, Vesic SA (1996). Pseudoepitheliomatous keratotic and micaceous balanitis. Case report and review of the literature. Uro Int.

[3] Reed SI, Abell E (1981). Pseudoepitheliomatous keratotic micaceous balanitis. Arch Dermatol.

[4] Perry D, Lynch PJ, Fazel N (2008). Pseudoepitheliomatous, keratotic, and micaceous balanitis: Case report and review of the literature. Dermatol Nurs.

[5] Gharpuray BM, Joshi PB, Palki HA, Lohalikar A (1990). Pseudoepitheliomatous, keratotic and micaeous balanitis. Indian J Dermatol Venerol Leprol.

[6] Subuddhi CL, Singh PC (1999). Pseudoepitheliomatous, keratotic and micaeous balanitis producing nail like lesions on the glans penis. Indian J Dermatol Venerol Leprol.

[7] Child FJ, Kim BK, Ganesan R, Southern SA, Herrington CS, Calonje E (2000). Verrucous carcinoma arising in pseudoepitheliomatous keratotic and micaceous balanitis, without evidence of human papilloma virus. Br J Dermatol.

[8] Querol Nasarre I, Córdoba Iturriagagoitia A, Castillo Jimeno JM, Ripa Saldias L, Monzón Muñoz FJ (1998). Keratotic pseudoepitheliomatous and micaceous balanitis. Arch Esp Urol.

[9] Jenkins D, Jakubovic HR (1988). Pseudoepitheliomatous, keratotic, micaceous balanitis. A clinical lesion with two histologic subsets: Hyperplastic dystrophy and verrucous carcinoma. J Am Acad Dermatol.

